# Epidemiologic profile of viral hepatitis B and C in North of Iran: results from PERSIAN Guilan Cohort Study (PGCS)

**DOI:** 10.1186/s13104-021-05474-2

**Published:** 2021-02-10

**Authors:** Fariborz Mansour-Ghanaei, Farahnaz Joukar, Mohammadreza Naghipour, Soheil Hassanipour, Sara Yeganeh, Masood Sepehrimanesh, Mohammad Fathalipour

**Affiliations:** 1 Caspian Digestive Diseases Research Center, Guilan University of Medical Sciences, Rasht, Iran; 2 Gastrointestinal and Liver Diseases Research Center, Guilan University of Medical Sciences, Rasht, Iran; 3 GI Cancer Screening and Prevention Research Center, Guilan University of Medical Sciences, Rasht, Iran; 4 University of Louisiana at Lafayette, Lafayette, LA USA; 5 Department of Pharmacology and Toxicology, Faculty of Pharmacy, Hormozgan University of Medical Sciences, Bandar Abbas, Iran

**Keywords:** Hepatitis B, Hepatitis C, PERSIAN cohort, Prevalence, Iran

## Abstract

**Objective:**

Hepatitis B (HB) and C (HC) are two severe viral infectious diseases with a deleterious impact on global health. This study aimed to evaluate the prevalence of HB and HC in the Prospective Epidemiological Research Studies of the Iranian Adults (PERSIAN) Guilan Cohort Study using immunological and molecular methods.

**Results:**

The blood samples were obtained from 10,520 enrolled participants. Complete biochemical and hematological tests, as well as urine analysis, were assessed. The presence of HBsAg, anti-HBs, anti-HBc, and anti-HCV antibodies for all participant and HBeAg and anti-HBe antibodies for HB-positive patients were evaluated. Moreover, HB genomic DNA and HC genomic RNA were extracted from serum samples of HB-positive patients. The real-time PCR assay was employed to quantify the gene copies of hepatitis B and C viruses. HC genotyping was also performed. The prevalence of HB and HC was 0.24% (95% CI 0.16–0.35) and 0.11% (95% CI 0.06–0.19), respectively. Rural participants were significantly more HB-positive than the urban people (P = 0.045), while males were significantly more HC-positive than the females (P = 0.013). The prevalence of HB and HC in this area were lower than those of other geographical locations of Iran, which may be due to different lifestyles or other unknown reasons.

## Introduction

Hepatitis B (HB) is a viral infection that affected the hepatic tissue and can cause other acute and chronic liver illnesses [1]. World Health Organization (WHO) statistics reported 240 million people contaminated with hepatitis B virus (HBV) in 2016 with HBsAg-positive test for at least 6 months [2]. The result of a modeling study in 2016 showed the global prevalence of HBsAg-positive HB was 3.9% (95% uncertainty interval [UI], 3.4–4.6) [3]. Finally, the highest rates of HB are reported in Africa and East Asia [4–7].

Hepatitis C (HC) is the leading cause of chronic liver disease, which can progress to chronic hepatocellular carcinoma with a high level of economic burden [8–14]. HB has silent epidemiology, and it is a primary blood-borne infection worldwide [15–17]. According to the latest global health statistics, 130–150 million people are infected with hepatitis C virus (HCV) [18], and 700,000 people die every year [19]. Between 1990 and 2013, the global deaths from viral hepatitis increased from 0.89 million (UI, 0.86–0.94) to 1.45 million (UI, 1.38–1.54) [20].

Therefore, Iran is classified as a low- to intermediate-prevalence area [21]. The last meta-analysis conducted on the general population of Iran showed the prevalence of HB was approximately 2.2% in 2016 [22].

Although specific populations, including hemophiliac and hemodialysis patients, are more prone to HB and HC [23], assessment of the prevalence of these diseases among the general population also is a critical issue. Considering the importance of HB and HC, the present study aimed to estimate the prevalence of these viral infections among the PERSIAN Guilan cohort study (PGCS) participants.

## Main text

### Methods

#### Participants

The PGCS is a part of Prospective Epidemiological Research Studies of the Iranian Adults (PERSIAN) cohort study, started in September 2014 in Someh’ E Sara (GPS coordinator Latitude: 37.308003 & Longitude: 49.315022), Guilan, Northern of Iran, and recruited both men and women aged 35–70 years, with the aim of subsequent, follow for 10 years to determine new diseases as well as identify the underlying genetic susceptibility factors for chronic diseases [24, 25].

This study was a cross-sectional study conducted on 10,520 people who had complete baseline information to diagnose HB and HC.

#### Sampling and biochemical assessments

The aseptic blood samples were collected from the cubital vein. The total number of white blood cells (WBC), red blood cells (RBC), platelet, lymphocyte, monocyte, and granulocytes were counted. The serum sample was harvested and stored at − 20 °C until the biochemical assessment. The concentration of hemoglobin (Hb) and level of hematocrit (HCT), mean corpuscular volume (MCV), mean corpuscular hemoglobin (MCH), mean corpuscular hemoglobin concentration (MCHC), red blood cell distribution width (RDW-CV and RDW-SD), Plateletcrit, mean platelet volume (MPV), platelet distribution width (PDW), lipid profile and liver function parameters were also evaluated. A urine sample was collected to measure the urine specific gravity (SG), pH, and creatinine level.

#### Virological assessments

The presence of hepatitis B surface antigen (HBsAg), anti-HBs antibody, anti-HBc antibody, and anti-HCV antibody was detected using Electrochemiluminescence (Cobas e 411, Roche, Germany). For positive patients, these four tests, along with the presence of HBeAg and anti-HBe antibodies, were measured again. The genomic DNA of HB was extracted from serum samples of HB-positive patients using a viral DNA extraction kit (QIAGEN, Germany). The genomic RNA of HC was also extracted from serum samples of HC-positive patients using a viral RNA extraction kit (Roche, Germany). The qPCR assay was carried out using a TaqMan-based commercially available kit (QIAGEN, Germany) to quantify the number of HB and HC gene copies based on the manufacturer’s instructions. The genotyping of HCV was performed using the Genotype Plus Real-TM kit (Sacace Biotechnologies, Italy).

#### Ethical statement

This study was approved by the Ethics Committee of Guilan University of Medical Sciences (Ethics code: IR.GUMS.REC. 1396.254).

#### Statistical analysis

The normality of data distribution evaluated using the Kolmogorov–Smirnov test. Thereafter, qualitative data were expressed as frequency and percentage, and their association with HB and HC statuses were analyzed using the Chi-square test. Quantitative data were presented as mean and standard deviation, and the differences between HB/HC-positive and negative groups were analyzed using two independent sample t-test. All statistical analysis was performed using SPSS version 23. The p-value of < 0.05 was considered as statistically significant. Garmin GPSMAP 78s was used for geographic distribution of participants.

### Results

Most participants were female (53.5%), rural (56.1%), married (97.2%), and primary educated (< 12 years) (72.1%) with history of smoking (75.2%) or alcohol consumption (85.3%). Besides, most of them had a history of hospitalization (80.6%) and surgery (63.3%) and had no transfusion (89.5%) or genital aphthous (98.8%). The demographic characteristics of the total participants were presented in Table 1.

**Table 1:** Mean and SD of quantitative variables plus frequency and percentage of qualitative variables in total participants and based on HB and HC statuses

Variables		HB			HC		
	Total (n = 10,520)	Positive (n = 25)	Negative (n = 10,495)	P value	Positive (n = 12)	Negative (n = 10,508)	P value
Age (year)	51.52 ± 8.90	54.48 ± 9.04	51.51 ± 8.90	0.095	54.08 ± 10.79	51.51 ± 8.89	0.317
BMI (kg/m^2^)	28.15 ± 5.82	26.32 ± 4.00	28.16 ± 5.82	0.114	26.49 ± 3.66	28.16 ± 5.83	0.323
Gender				0.078			0.013
Male	4887 (46.5)	16 (0.3)	4871 (99.7)		9 (0.2)	4878 (99.8)	
Female	5633 (53.5)	9 (0.2)	5624 (99.8)		3 (0.1)	5630 (99.9)	
Habitat				0.045			0.108
Urban	4619 (43.9)	6 (0.1)	4613 (99.9)		7 (0.1)	4612 99.9)	
Rural	5901 (56.1)	19 (0.3)	5882 (99.7)		5 (0.1)	5896 (99.9)	
Marital status				0.490			0.647
Married	10,224 (97.2)	25 (0.2)	10,199 (99.8)		12 (0.1)	10,212 (99.9)	
Single	296 (2.8)	0 (0)	296 (100)		0 (0)	296 (100)	
Education				0.301			0.673
Primary (< 12 years)	7590 (72.1)	21 (0.3)	7569 (99.7)		10 (0.1)	7580 (99.9)	
Diploma (12 years)	2284 (21.7)	4 (0.2)	2280 (99.8)		2 (0.1)	2282 (99.9)	
Academic (> 12 years)	646 (6.1)	0 (0)	646 (100)		0 (0)	646 (100)	
Smoking				0.861			0.943
Yes	2577 (24.5)	5 (0.2)	2572 (99.8)		3 (0.1)	2574 (99.9)	
No	7908 (75.2)	20 (3)	7888 (99.7)		9 (0.1)	7899 (99.9)	
Not-sure	7 (0.1)	0 (0)	7 (100)		0 (0)	7 (100)	
Alcohol consumption				0.497			0.900
Yes	1515 (14.4)	4 (0.3)	1511 (99.7)		2 (0.1)	1513 (99.9)	
No	8977 (85.3)	21 (0.2)	8956 (99.8)		10 (0.1)	8967 (99.9)	
Surgery				0.939			0.512
Yes	6637 (63.3)	16 (0.2)	6621 (99.8)		9 (0.1)	6628 (99.9)	
No	3855 (36.7)	9 (0.2)	3848 (99.8)		3 (0.1)	3852 (99.9)	
Hospitalization				0.561			0.653
Yes	8456 (80.6)	19 (0.2)	8437 (99.8)		10 (0.1)	8446 (99.9)	
No	2036 (19.4)	6 (0.3)	2030 (99.7)		2 (0.1)	2034 (99.9)	
Transfusion				0.703			0.970
Yes	1001 (9.5)	4 (0.4)	997 (99.6)		1 (0.1)	1000 (99.9)	
No	9395 (89.5)	21 (0.2)	9374 (99.8)		11 (0.1)	9384 ()	
Not know	95 (0.9)	0 (0)	95 (100)		0 (0)	95 (100)	
Genital aphthous				0.732			0.828
Yes	130 (1.2)	0 (0)	130 (100)		0 (0)	130 (100)	
No	10,362 (98.8)	25 (0.2)	10,337 (99.8)		12 (0.1)	10,350 (99.9)	

**Fig. 1:**
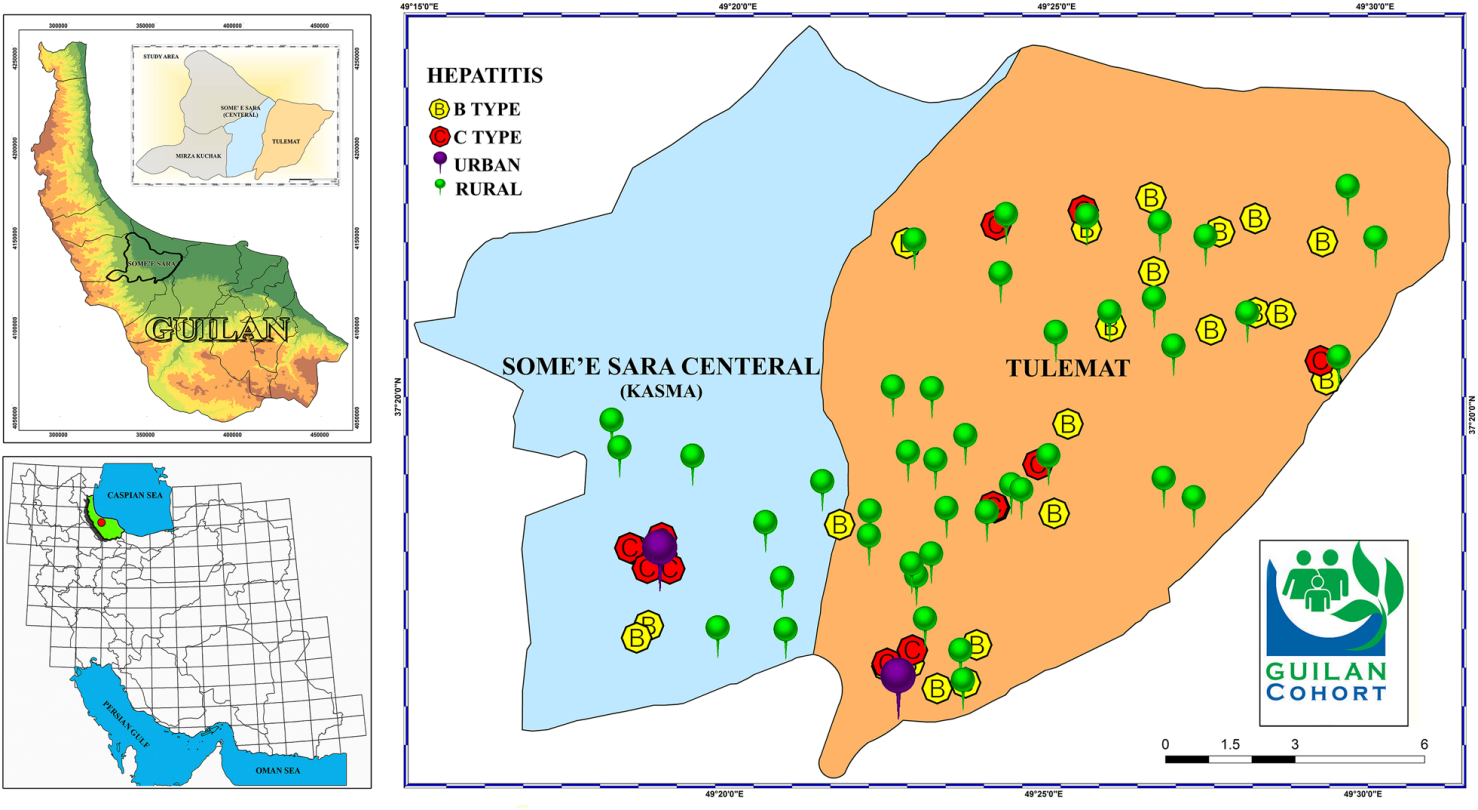
Geographic distribution of HB and HC positive patients (The map depicted in figure is our own work)

According to the qPCR assay, the overall prevalence of HB and HC was 0.24% (CI 0.16–0.36) (n = 25) and 0.11% (CI 0.06 to 0.19%) (n = 12), respectively. The geographic distribution of HB-positive and HC-positive patients based on gender was presented in Fig. 1. The prevalence of HB and HC in Tulmat (0.38 and 0.14%, respectively) are higher compared to the Central part of Someh’ E Sara (0.06 and 0.08%, respectively). Rural participants had a significantly higher prevalence of HB than the urban population (P = 0.045). Male individuals had a significantly higher prevalence of HC than female participants (P = 0.013). No further associations were detected between other variables and the prevalence of HB and HC. Moreover, the prevalence of drug abusers among rural HB-positive patients (n = 13) is higher compared to urban HB-positive patients (n = 1) (p < 0.05).

HB-positive patients had significantly lower platelet count (P = 0.043), RDW-CV (P = 0.023), cholesterol (P = 0.033), LDL-C (P = 0.043), and LDL-C: HDL-C ratio (P = 0.002) compared to HB-negative patients. On the other hand, HC-positive patients had significantly higher MCH (P = 0.036), MCHC (P = 0.047), AST (P = 0.032), ALT (P = 0.030), and HDL-C (P = 0.039) and significant lower LDL-C (P = 0.028) and LDL-C: HDL-C ratio (P = 0.001) compared to HC-negative individuals. The complete blood and urine analyses of all participants were presented in Table 2.

**Table 2:** Comparison of blood and urine analysis based on HB and HC statuses

Variables	HB			HC		
	Positive (n = 25)	Negative (n = 10,495)	P value	Positive (n = 13)	Negative (n = 10,507)	P value
Blood analysis						
WBC (/mm^3^)	6.49 ± 1.22	7.08 ± 1.73	0.090	6.13 ± 2.27	7.08 ± 1.73	0.058
RBC (× 1000/mm^3^)	4.89 ± 0.47	4.90 ± 0.56	0.969	4.65 ± 0.65	4.90 ± 0.55	0.125
Hb (g/dL)	14.02 ± 1.47	13.82 ± 1.57	0.514	13.97 ± 1.82	13.82 ± 1.57	0.738
Hct (%)	41.95 ± 3.72	41.33 ± 4.15	0.459	41.05 ± 4.71	41.33 ± 4.14	0.812
MCV (fl)	86.20 ± 8	84.88 ± 7.57	0.384	88.88 ± 8.66	84.87 ± 7.56	0.066
MCH (pg)	28.82 ± 3.27	28.39 ± 3.05	0.481	30.23 ± 3.42	28.39 ± 3.05	0.036
MCHC (g/dL)	33.38 ± 1	33.39 ± 1.01	0.964	33.97 ± 0.90	33.39 ± 1.01	0.047
Platelet (/mm^3^)	227.92 ± 65.36	251.91 ± 59.29	0.043	234.75 ± 74.99	251.90 ± 59.31	0.317
Lymphocyte %)	36.07 ± 7.12	38.71 ± 9.23	0.269	36.09 ± 9.42	38.71 ± 9.22	0.394
Monocyte (%)	1.06 ± 0.29	1.23 ± 0.70	0.340	1.62 ± 1.19	1.23 ± 0.70	0.093
Granulocytes (%)	62.87 ± 7.15	60.06 ± 9.43	0.249	62.29 ± 9.95	60.06 ± 9.42	0.478
RDW-CV (%)	12.01 ± 0.50	12.57 ± 0.98	0.023	12.75 ± 1.58	12.57 ± 0.98	0.550
RDW-SD (fl)	41.51 ± 3.88	42.29 ± 3.48	0.374	45.93 ± 9.90	42.27 ± 3.45	0.273
Plateletcrit (× 1000/µl)	0.21 ± 0.05	0.21 ± 0.05	0.831	0.19 ± 0.06	0.21 ± 0.05	0.198
MPV (fl)	8.23 ± 0.51	8.16 ± 0.71	0.706	8.41 ± 0.69	8.16 ± 0.71	0.274
PDW (fl)	16.23 ± 1.59	16.70 ± 1.04	0.072	16.88 ± 1.12	16.70 ± 1.04	0.574
Glucose (mg/dL)	102.28 ± 52.78	104.57 ± 37.14	0.758	95.58 ± 13.73	104.58 ± 37.22	0.402
BUN (mg/dL)	14.10 ± 4.09	13.37 ± 3.53	0.304	14.93 ± 5.65	13.37 ± 3.50	0.123
Creatinine (mg/dL)	0.95 ± 0.15	0.89 ± 0.17	0.084	0.98 ± 0.25	0.89 ± 0.16	0.060
Triglyceride (mg/dL)	149.12 ± 236.54	160.31 ± 102.79	0.815	102.25 ± 42.39	160.37 ± 103.37	0.052
Cholesterol (mg/dL)	176.20 ± 32.35	192.85 ± 38.98	0.033	172.50 ± 26.75	192.86 ± 38.97	0.070
HDL (mg/dL)	49.20 ± 10.25	48.39 ± 10.99	0.711	59.58 ± 16.59	48.38 ± 10.97	0.039
LDL (mg/dL)	99.88 ± 25.21	112.88 ± 32.07	0.043	92.50 ± 22.20	112.89 ± 32.05	0.028
LDL:HDL	2.06 ± 0.52	2.42 ± 0.79	0.002	1.67 ± 0.62	2.42 ± 0.79	0.001
AST (IU/L)	19.20 ± 7.82	19.06 ± 8.43	0.935	46.58 ± 39.00	19.02 ± 8.25	0.032
ALT (IU/L)	15.72 ± 8.26	18.82 ± 13.54	0.253	38.25 ± 27.10	18.77 ± 13.43	0.030
ALP (IU/L)	194.00 ± 52.32	207.03 ± 59.88	0.277	215.75 ± 55.11	206.98 ± 59.89	0.612
GGT (IU/L)	18.37 ± 6.40	25.05 ± 20.80	0.108	59.17 ± 73.63	25.00 ± 20.62	0.136
Vit D_3_ (ng/ml)	25.01 ± 14.88	21.77 ± 12.39	0.201	26.94 ± 13.97	21.77 ± 12.40	0.149
Urine analysis						
SG	1.02 ± 0.01	15.18 ± 119.12	0.552	1.02 ± 0.01	15.18 ± 119.14	0.680
pH	5.88 ± 0.89	5.83 ± 0.88	0.760	5.75 ± 0.75	5.83 ± 0.88	0.761
Creatinine (mg/dL)	134.17 ± 71.03	140.36 ± 77.30	0.749	136.43 ± 79.52	140.36 ± 77.32	0.872

Most HB-positive patients (52%) had < 300 gene copies/ml of HBV. While most HC-positive patients (58.4%) had 10^5^–10^6^/ml gene copies of HCV. The most detected HCV genotype was 2a (58.33%) compared to 1a (25.00%) and 1a and 2a (16.67%). First-degree relatives of all HC-positive patients were also checked for HC using qPCR. Only a child had an HC infection with a genotype similar to that of her mother (1a).

### Discussion

In the present study, the prevalence of HB and HC among the participants of the PERSIAN Guilan Cohort Study (PGCS) were 0.24 and 0.11%, respectively. Moreover, rural participants were significantly more HB-positive, while male individuals were significantly more HC-positive. HB-positive patients had significantly lower platelet count, RDW-CV, cholesterol, LDL-C, and LDL-C: HDL-C ratio and HC-positive patients had significantly higher MCH, MCHC, AST, ALT, and HDL, and significant lower LDL-C and LDL-C: HDL-C ratio compared to related negative individuals.

The prevalence of HB and HC is very different worldwide, according to geographical region and demographic factors. In 2015, it has been reported that HB seroprevalence was 8.83% (CI 0.48–22.38) in African region, 0.81% (CI 0.20–13.55) in Americas region, 3.01% (CI 0.67–14.77) in Eastern Mediterranean region, 2.06% (CI 0.01–0.32) in European region, 1.90% (CI 0.82–6.42) in South East Asian region, and 5.26% (CI 0.37–22.70) in Western Pacific region [26]. Also, there is much diversity in the prevalence of HB between different states/provinces of each country. Since 2006 when the national vaccination program for Iranian people born after 1993 was started and continued, an obvious decrease in the prevalence of HB was seen [27]. Therefore, Iran is classified as a low- to an intermediate- prevalence areas [21]. Although the detected HB infection rate is lower than the reported pooled prevalence of HB in Iran among the general population (2.2%) in 2016 [22]. It is approximately similar to our previous report about volunteer blood donors (0.45–0.48%) [28] and to reported rates from Karaj (0.4%) [29], Kermanshah (0.7%) [30] and Kurdistan (0.8%) [31]. Also, our reported rate of HB infection is lower than those reported from Birjand (1.6%) [32], Tehran, Golestan, Hormozgan (2.6%) [33], and Nahavand (2.3%) [34]. In addition, some population sub-groups are more likely susceptible for infection with HB. For instance, in Guilan province, 71.3% of hemophiliacs [35] and 0.38 to 3.8% of hemodialysis patients [36–38] were HB-positive patients. We found that men are more HB-positive than women (16 vs. 9 cases), which is similar to previous reports from Iran about a higher prevalence of HB infection in men [22, 39]. Furthermore, the prevalence of HB and HC in Tulemat is higher than in other geographical areas of Someh’ E Sara. It might be related to the high number of drug abusers in this area compared to other areas. Based on the subgroup analysis, the prevalence of drug abusers among rural HB-positive patients is higher compared to urban HB-positive patients.

The pooled prevalence of HC was reported 0.3, 6.2, and 32.1% for general, intermediate- and high-risk Iranian populations, respectively [40]. Again, diversities between different cities/provinces and subgroups are seen. It has been reported that all healthy adults of Isfahan and Mashhad, blood donors of Tehran, Ardabil, and Ahvaz, infertile male of Tehran, and male blood donors of Tabriz were HC-negative [40]. Our detected prevalence of HC (0.1%) is lower than the pooled prevalence of HC among the general population of Iran (0.3%) [40] and is differed from the previous report from Rasht (0.03%) and Guilan (0.32%) [28]. Also, our detected HC prevalence is lower than other reported prevalence from the Northern provinces of Iran. The prevalence of HC was 0.48% in Babol and 0.18 to 1.00% in Golestan. However, Zamani et al. reported a similar prevalence of HC (0.08%) in the general population of Mazandaran province. A higher prevalence of HC among males was also reported previously from Kerman, Zahedan, and Kavar. However, Ghadir et al. reported that females were more HC-positive compared to males in the general population of Golestan [40, 41]. The finding of one infected woman, whose her daughter also was HC-positive and both had the same HC genotype, highlighted the precise role of interfamilial transmission and confirmed the significant role of close relatives [42].

Although we detected no significant associations between most of the demographic variables and the prevalence of HB and HC, it seems that different demographic features of the population in different regions are the most important reasons for these differences in the prevalence of HB and HC. Based on Baig’s study, the male to female ratio of the prevalence of HB increased during the reproductive years. There might be a protective effect of estrogen on the hepatocytes against the development of chronic liver disease [43]. In Zeng et al. study, married people had the highest prevalence of HBsAg [44]. On the other hand. Ataei et al. demonstrated no statistical difference observed in terms of marital status in Isfahan province, but males (OR = 3.79) had a higher prevalence of HB than women [45].

Regarding biochemical analysis, we found some significant differences. A decrease in LDL-C level and subsequently LDL-C: HDL-C ratio in HB- and HC-positive patients is interesting. These are in line with those reported recently as significant hypolipidemia in patients with HB [46] and HC [47]. Lower platelet count in HB-positive was also reported previously [48]. Both HB and HC potentially influenced the liver tissue, and the changes in biochemical and hematological parameters could be related to these changes in the hepatic functions.

### Conclusion

In summary, we found lower HB and HC prevalence compared to other regions of Iran. Compared to previous reports from our province, Guilan, the HB and HC prevalence also decreased. It may be due to the preventive strategy or increase of the medical knowledge of peoples, which must be evaluated in further studies.

### Limitation

The limitation of our investigation was the study of a specific age group and did not include the high-risk population, such as young people, sex workers, and intravenous drug abusers.

## Data Availability

The study protocol and the datasets analyzed are available from the corresponding author upon request.

## Abbreviations

HB: Hepatitis B
HC: Hepatitis C
HBcAg: Hepatitis core antigen
HBsAg: Hepatitis B surface antigen
